# Supplementary material for: αV-class integrins exert dual roles on α5β1 integrins to strengthen adhesion to fibronectin
Source: Nat Commun. 2017 Jan 27;8:14348. doi: 10.1038/ncomms14348 (PMC5290147; doi:10.1038/ncomms14348)
Supplement: Supplementary Information — Supplementary Figures, Supplementary Table and Supplementary Note [file ncomms14348-s1.pdf]

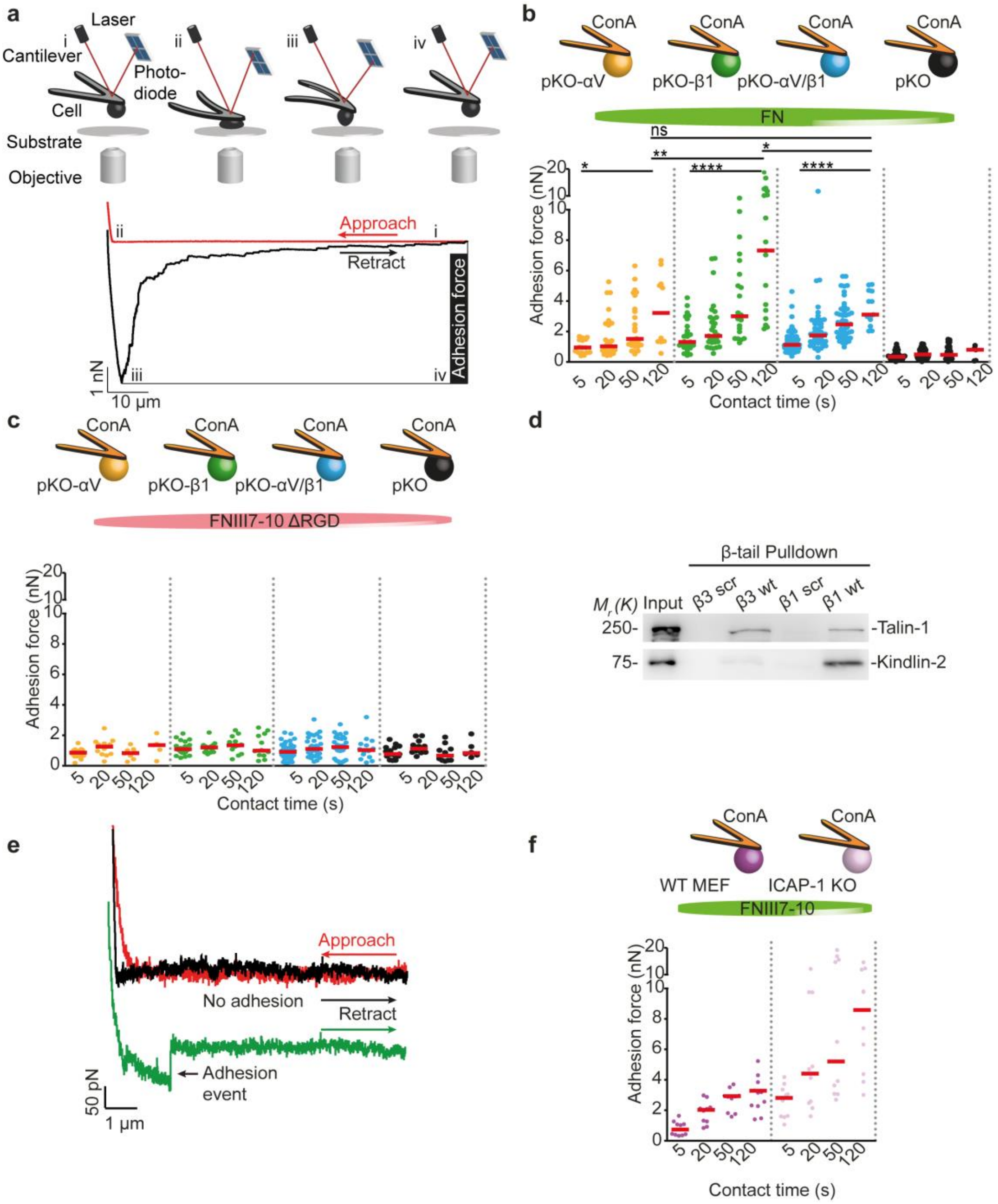

Supplementary Figure 1. Differential contribution α5β1 and αV-class integrins to the early adhesion of mouse kidney fibroblasts. (**a**) AFM-based single-cell force spectroscopy (SCFS) to characterize the adhesion of fibroblasts. (i) A single fibroblast is immobilized on a concanavalin A (ConA)-functionalized cantilever for 7-10 minutes. (ii) The cantilever-attached fibroblast is approached to a functionalized substrate until reaching a preset contact force (≈ 2 nN). Then, the cantilever height is maintained for a defined contact time. (iii-iv) Subsequently, the cantilever is retracted to separate fibroblast and substrate. The force deflecting the cantilever and the distance travelled by the cantilever is displayed in a force-distance curve. During retracting the cantilever, the adhesion force is measured. (**b,c**) Adhesion force of four different fibroblast lines, expressing either αV-class integrins (pKO-αV, yellow), or

α5β1 integrins (pKO-β1, green), or α5β1 and αV-class integrins (pKO-αV/β1, blue), or pan-integrin knockout (pKO, black) was characterized by SCFS. The adhesion of fibroblasts bound to ConA-functionalized cantilevers was measured to substrates coated by (**b**) full-length FN and (**c**) RGD-deleted FN fragments FNIII7–10ΔRGD for contact times ranging from 5–120 s. Dots show adhesion forces of single fibroblasts ($n \geq 10$ for each condition) and red bars their median. Statistical significances were analyzed by two-tailed Mann-Whitney U-tests (****, $P < 0.0001$; ***, $P < 0.001$; **, $P < 0.01$; *, $P < 0.05$; *ns,* $P \geq 0.05$). (**d**) Western blot showing talin-1 and kindlin-2, binding to biotinylated β1- and β3-integrin tail peptides. Peptides with scrambled amino-acid sequences (β1 scr tail; β3 scr tail) were used as negative controls. WT, wild-type. Input, whole wild-type (WT) pKO-αV/β1 fibroblast lysate. (**e**) Force-distance curves representing the binding of single integrins of pKO-αV/β1 fibroblasts to a FN-coated substrate. To record single integrin binding events with SCFS, fibroblasts were approached to the substrate (red curve) at minimal contact force (200 pN) and contact time (≈ 100ms) and then retracted (black/green curve). The green force-distance curve shows a single adhesion event while the black curve shows no adhesion event. (**f**) Adhesion forces of WT mouse embryonic fibroblasts (WT MEF, left panel) and ICAP-1 deficient MEF (ICAP-1 KO, right panel) to FNIII7-10-coated substrates. Fibroblasts were attached to ConA-coated cantilevers. Dots show adhesion forces of single fibroblasts ($n \geq 10$ for each condition) and red bars their median.

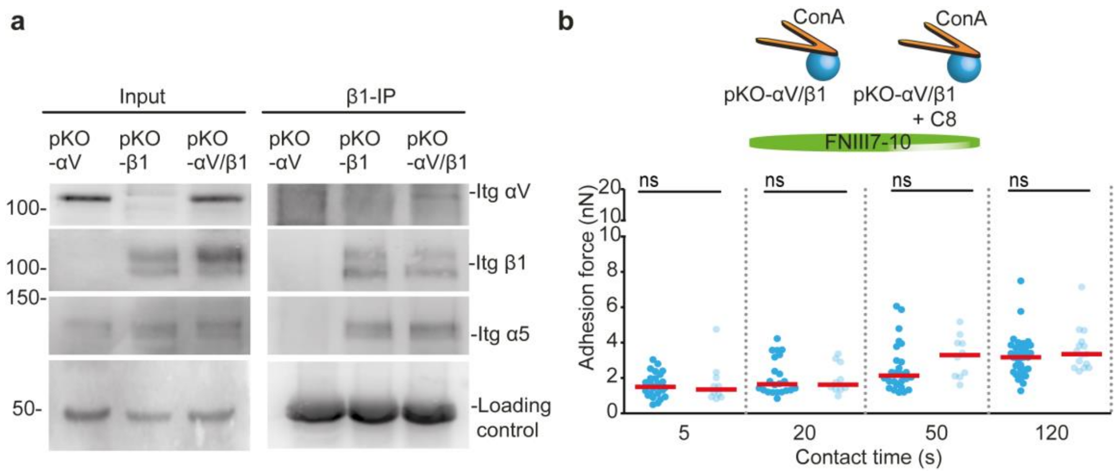

Supplementary Figure 2. αVβ1 integrins do not contribute to fibroblast adhesion to FN. (**a**) Total cell lysates (Input panel) and immunoprecipitated β1 integrin (β1-IP panel) were immunoblotted for αV, α5 and β1 integrins. We observed comparable amounts of αV-class and α5β1 integrins in all fibroblasts lines. The αVβ1 heterodimer was also detected but at considerably lower amounts compared to αV-class and α5β1 integrins in pKO-αV/β1 fibroblasts. (**b**) αVβ1 integrins do not play vital roles in early fibroblast adhesion to FNIII7–10. pKO-αV/β1 fibroblasts were incubated with C8 inhibitor for 30 minutes, then attached to ConA-coated cantilevers and finally approached to FNIII7–10 for contact times ranging from 5–120 s. The adhesion of C8 inhibitor treated fibroblasts was comparable to untreated. Dots show adhesion forces of single fibroblasts (n ≥ 10 for each condition) and red bars their median. Statistical significances were analyzed with two-tailed Mann-Whitney U-tests (****, $P < 0.0001$; ***, $P < 0.001$; **, $P < 0.01$; *, $P < 0.05$; *ns*, $P \geq 0.05$).

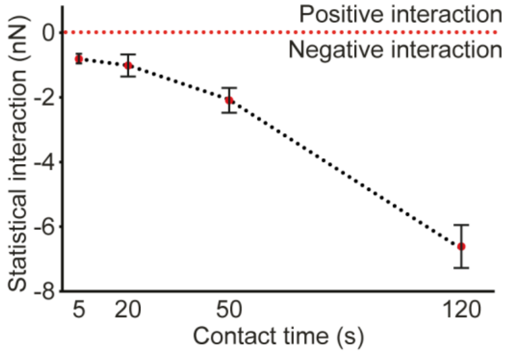

Supplementary Figure 3. Statistical interaction reveals that αV-class integrins suppress α5β1 integrins from binding to FN. Displayed is the statistical interaction measured as the deviation of the adhesion force from the expected additive effect of αV-class and α5β1 integrins when present together relative to the knockout pKO fibroblasts, for each contact time. The statistical interactions determined (see Methods) are significantly different from zero (and negative) at all four contact times, clearly showing a negative relationship with respect to adhesion force between αV-class and α5β1 integrins when present together. Red circles show mean values and error bars the s.e.m. ($n = 100$) The R-code used for the statistical analysis is shown in the Supplementary data.

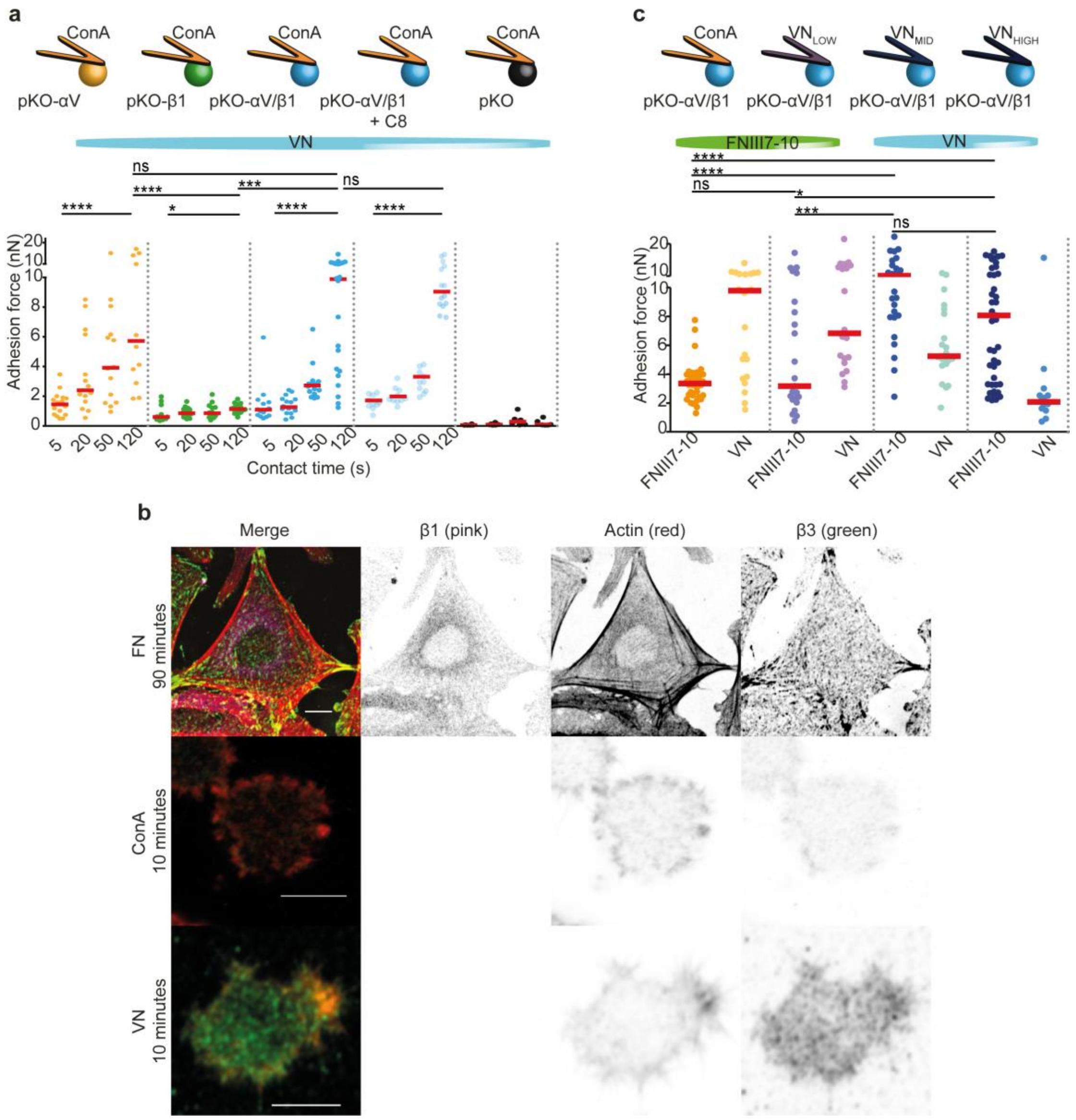

Supplementary Figure 4. αV-class integrins bind and cluster on VN. (**a**) Adhesion force of fibroblast lines expressing either αV-class integrins (pKO-αV, yellow), or α5β1 integrins (pKO-β1, green), or α5β1 and αV-class integrins (pKO-αV/β1, blue untreated and light blue C8 inhibitor treated) or pan-integrin knockout (pKO, black) contacted to VN-substrate for times ranging from 5–120 s. All fibroblasts were attached to ConA-functionalized cantilevers. (**b**) Immunofluorescence of pKO-αV/β1 fibroblasts seeded on VN (5 μg ml^{-1} VN diluted in ConA)-, ConA-, or FN-functionalized substrates. Fibroblasts adhering to VN, ConA for 10 minutes and to FN for 90 minutes were stained for αV-class integrin (green), actin (red) and β1 integrin (pink) using β3 integrin specific antibodies for the detection of αVβ3 integrins, β1 integrin antibody (Methods) and phalloidin. pKO-αV/β1 fibroblasts adhering to VN for 10 minutes mimic the condition of VN-stimulated fibroblasts, wherein fibroblast adhere for 7–10 minutes to VN-functionalized cantilevers. Clustered αV-class and α5β1 integrins on FN for 90 minutes were referred to as a positive control. Scale bars, 10 μm. (**c**) Fibroblasts enhance adhesion to FN upon VN-stimulation in a concentration-dependent manner. pKO-αV/β1 fibroblasts were attached to cantilevers coated either with ConA only or

with VN concentrations of 0.5 µg ml^{-1} (VN_{LOW}), 5 µg ml^{-1} (VN_{MID}, used in experiments above), both diluted in ConA, or 50 µg ml^{-1} (VN_{HIGH}). Single fibroblasts were attached to the cantilever for 7–10 minutes, then approached to the FNIII7–10-coated substrate for 120 s and finally retracted to measure the adhesion force. At 120 s contact time to FNIII7–10-coated substrates, VN_{LOW}-stimulated pKO-αV/β1 fibroblasts established adhesion forces comparable to fibroblasts attached to ConA-coated cantilevers, whereas VN_{MID}- and VN_{HIGH}-stimulated pKO-αV/β1 fibroblasts considerably strengthened adhesion to FNIII7–10. These experiments show that increasing the VN-density/coating on the cantilever more αV-class integrins were recruited, which were thus unavailable for establishing adhesion to the VN-coated substrate located at the opposite side of the cell. Dots show adhesion forces of single fibroblasts ($n \geq 10$ for each condition) and red bars their median. Statistical significances were calculated with two-tailed Mann-Whitney U-tests (****, $P < 0.0001$; ***, $P < 0.001$; **, $P < 0.01$; *, $P < 0.05$; *ns,* $P \geq 0.05$).

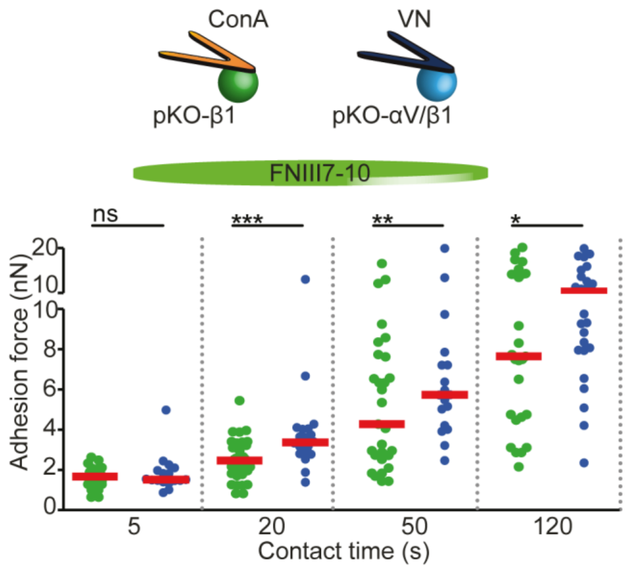

Supplementary Figure 5. αV-class integrins signal to regulate fibroblast adhesion to FN *via* β1 integrins. Adhesion forces of pan-integrin knockout (pKO) fibroblasts rescued with α5β1 integrins attached to ConA-coated cantilevers (pKO-β1, green) and α5β1 and αV-class integrins attached to VN-coated cantilevers (pKO-αV/β1, blue). VN-coated cantilevers were coated by 5 μg ml^{-1} VN diluted in ConA. Dots show adhesion forces of single fibroblasts ($n \geq 10$ for each condition) and red bars their median. Statistical significances were calculated with two-tailed Mann-Whitney U-tests. ****, $P < 0.0001$; ***, $P < 0.001$; **, $P < 0.01$; *, $P < 0.05$; ns, $P \geq 0.05$.

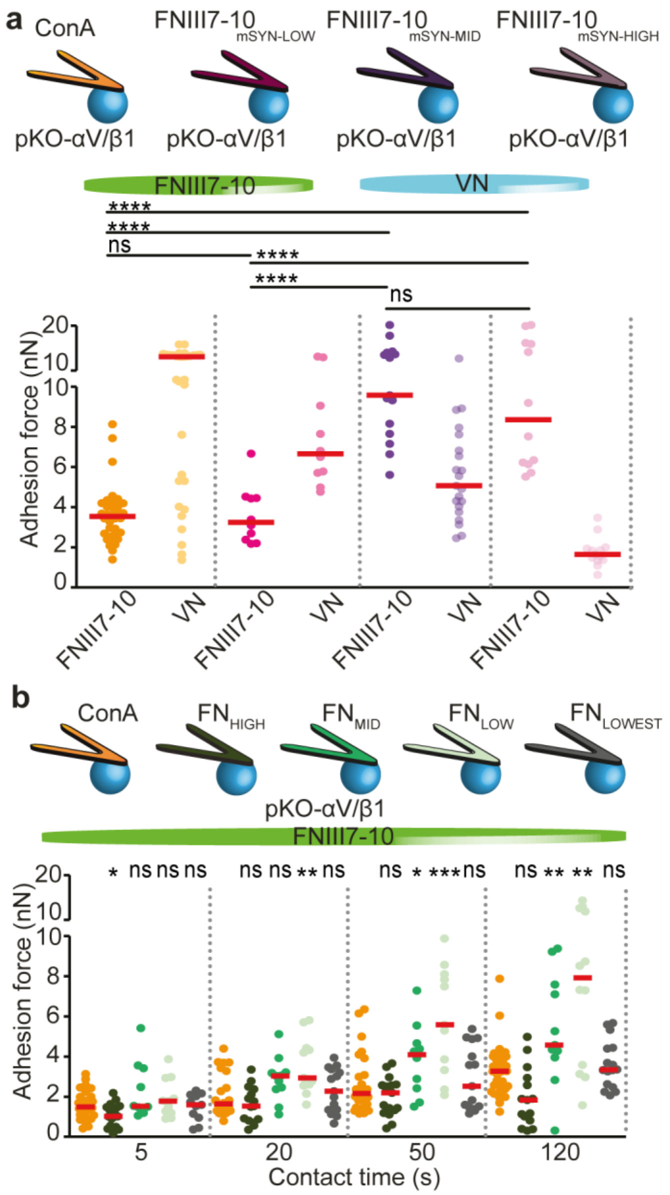

Supplementary Figure 6. FN can activate αV-class integrins to stimulate fibroblasts for strengthening adhesion to FN. FNIII7–10-mediated adhesion forces of pKO-αV/β1 fibroblasts attached either to (**a**) FN-fragment FNIII7–10 with mutated synergy site (FNIII7–10$_{mSYN}$) (**b**) or full length FN. pKO-αV/β1 fibroblasts either attached to a ConA-functionalized cantilever or to cantilevers functionalized with different FNIII7–10$_{mSYN}$ or FN concentrations (FNIII7-10$_{mSYN\text{-}HIGH}$/ FN$_{HIGH}$ 50 µg ml^{-1}, FNIII7-10$_{mSYN\text{-}MID}$/ FN$_{MID}$ 5 µg ml^{-1}, FNIII7-10$_{mSYN\text{-}LOW}$/ FN$_{LOW}$ 0.5 µg ml^{-1}, FN$_{LOWEST}$ 0.05 µg ml^{-1}). Prior to adhesion measurements, single fibroblasts were incubated on the cantilever for 7–10 minutes and then approached to the FNIII7-10-coated substrate for 120 s in (**a**) and for contact times as indicated in (**b**). Finally, single fibroblasts were retracted to measure the adhesion force. Dots show adhesion forces of single fibroblasts (n ≥ 10 for each condition) and red bars their median. Statistical significances were analyzed with two-tailed Mann-Whitney U-tests (****, $P < 0.0001$; ***, $P < 0.001$; **, $P < 0.01$; *, $P < 0.05$; *ns,* $P \geq 0.05$).

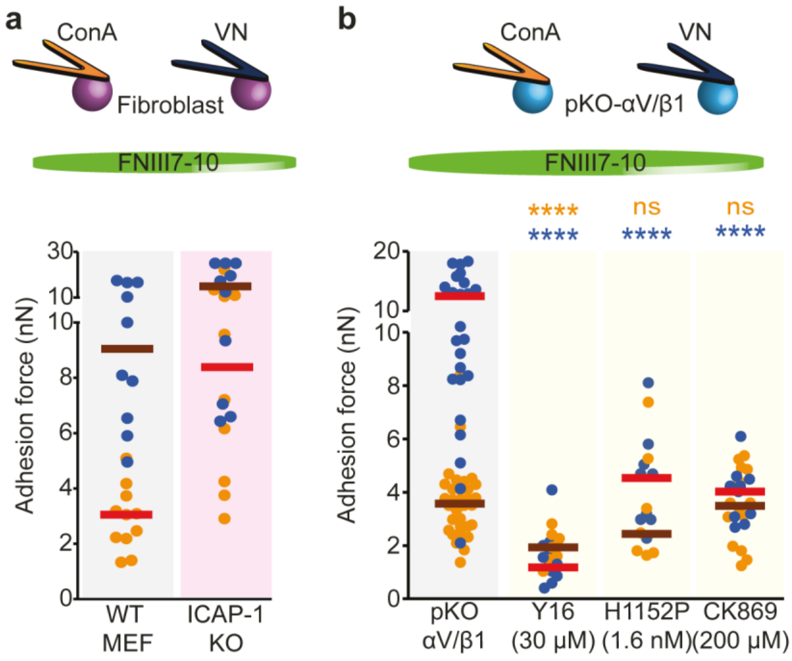

Supplementary Figure 7. Contribution of integrin associated proteins to the crosstalk between α5β1 and αV-class integrins. (**a**) Adhesion forces of ConA-attached (yellow) and VN-stimulated (blue) WT mouse embryonic fibroblasts (WT MEF, first panel) and ConA- and VN-attached ICAP-1 deficient MEFs (ICAP-1 KO, second panel) to FNIII7-10 after 120 s are shown. (**b**) After incubating single pKO-αV/β1 fibroblasts to either ConA (yellow)- of VN (blue)-coated cantilevers, their adhesion forces after 120 s contact time to FNIII7–10 were measured. 30 minutes prior to the experiments, the pKO-αV/β1 fibroblasts were incubated with the chemical inhibitors at given concentrations. For VN-stimulating fibroblasts, cantilevers were coated by 5 µg ml^{-1} VN diluted in ConA. Dots show adhesion forces of single fibroblasts ($n \geq 10$ for each condition) and red bars their median. Statistical significance was determined to compare non-stimulated and stimulated adhesion for each condition by with two-tailed Mann-Whitney U-tests (****, $P < 0.0001$; ***, $P < 0.001$; **, $P < 0.01$; *, $P < 0.05$; *ns*, $P \geq 0.05$).

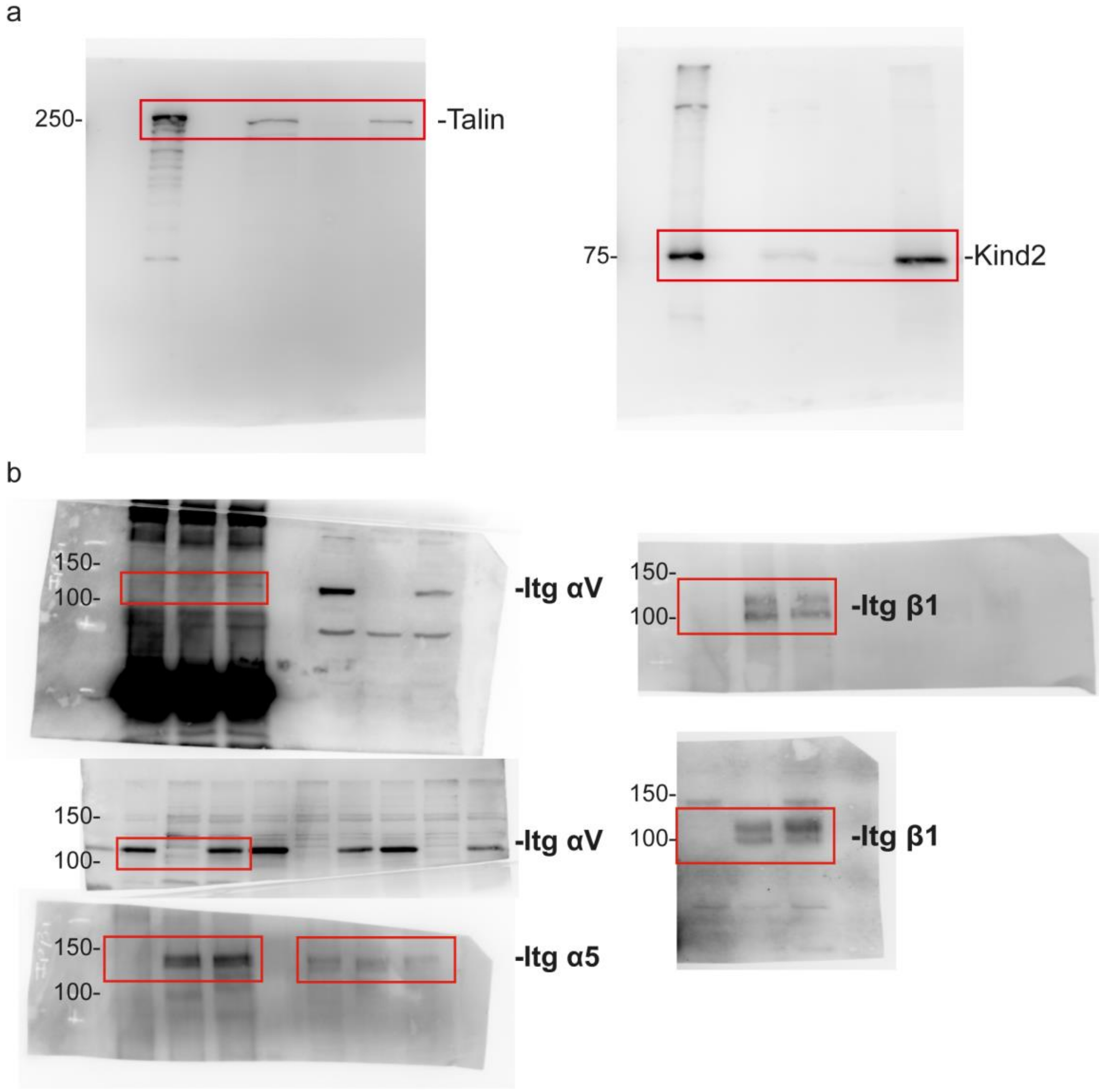

Supplementary Figure 8. Uncropped images of western blots shown in Supplementary Fig. 1d. and Supplementary Fig. 2a. (**a**) Western blots showing talin-1 and kindlin-2, binding to biotinylated β1- and β3-integrin tail peptides. Peptides with scrambled amino-acid sequences (β1 scr tail; β3 scr tail) were used as negative controls. WT, wild-type. Input, whole wild-type (WT) pKO-αV/β1 fibroblast lysate. (**b**) Total cell lysates (Input panel) and immunoprecipitated β1 integrin (β1-IP panel) were immunoblotted for αV, α5 and β1 integrins. We observed comparable amounts of αV-class and α5β1 integrins in all fibroblasts lines.

	pKO-αVβ1 non-stimulated	pKO-αVβ1 VN-stimulated	pKO-αV non-stimulated	pKO-αV VN-stimulated	pKO-β1 non-stimulated	pKO-β1 VN-stimulated
pKO-αVβ1 non-stimulated	ns	***	ns	**	***	***
pKO-αVβ1 VN-stimulated	***	ns	***	***	***	***
pKO-αV non-stimulated	ns	***	ns	**	***	***
pKO-αV VN-stimulated	**	***	**	ns	*	*
pKO-β1 non-stimulated	***	***	***	*	ns	ns
pKO-β1 VN-stimulated	***	***	***	*	ns	ns

Supplementary Table 1. Statistical analysis comparing the paxillin-GFP-intensity detected by TIRF microscopy in Fig. 4. Two-tailed Mann-Whitney tests were applied to determine significant differences between the paxillin-GFP-intensity among different conditions (n≥10 for each condition) as shown in Fig. 4. ****, $P < 0.0001$; ***, $P < 0.001$; **, $P < 0.01$; *, $P < 0.05$; *ns*, $P \geq 0.05$.

Supplementary Note 1

R-code for statistical analysis in Supplementary Fig. 3

```
# load data:
D  <- read.table(
  "FN.csv",
  header=T,
  sep=","
);
colnames(D) <- c(
  "a5", "a20", "a50", "a120",
  "b5", "b20", "b50", "b120",
  "ab5", "ab20", "ab50", "ab120",
  "null5", "null20", "null50", "null120"
);
a5 <- D[is.na(D[1]) == FALSE, 1];
a20 <- D[is.na(D[2]) == FALSE, 2];
a50 <- D[is.na(D[3]) == FALSE, 3];
a120 <- D[is.na(D[4]) == FALSE, 4];
b5 <- D[is.na(D[5]) == FALSE, 5];
b20 <- D[is.na(D[6]) == FALSE, 6];
b50 <- D[is.na(D[7]) == FALSE, 7];
b120 <- D[is.na(D[8]) == FALSE, 8];
ab5 <- D[is.na(D[9]) == FALSE, 9];
ab20 <- D[is.na(D[10]) == FALSE, 10];
ab50 <- D[is.na(D[11]) == FALSE, 11];
ab120 <- D[is.na(D[12]) == FALSE, 12];
null5 <- D[is.na(D[13]) == FALSE, 13];
null20 <- D[is.na(D[14]) == FALSE, 14];
null50 <- D[is.na(D[15]) == FALSE, 15];
null120 <- D[is.na(D[16]) == FALSE, 16];

# bootstrap samples:
n = 100;
null <- matrix(ncol = 4, nrow = n);
null[,1] <- sample(null5, n, replace = TRUE);
```

```
null[,2] <- sample(null20, n, replace = TRUE);
null[,3] <- sample(null50, n, replace = TRUE);
null[,4] <- sample(null120, n, replace = TRUE);
a <- matrix(ncol = 4, nrow = n);
a[,1] <- sample(a5, n, replace = TRUE);
a[,2] <- sample(a20, n, replace = TRUE);
a[,3] <- sample(a50, n, replace = TRUE);
a[,4] <- sample(a120, n, replace = TRUE);
b <- matrix(ncol = 4, nrow = n);
b[,1] <- sample(b5, n, replace = TRUE);
b[,2] <- sample(b20, n, replace = TRUE);
b[,3] <- sample(b50, n, replace = TRUE);
b[,4] <- sample(b120, n, replace = TRUE);
ab <- matrix(ncol = 4, nrow = n);
ab[,1] <- sample(ab5, n, replace = TRUE);
ab[,2] <- sample(ab20, n, replace = TRUE);
ab[,3] <- sample(ab50, n, replace = TRUE);
ab[,4] <- sample(ab120, n, replace = TRUE);

# slope:
slope <- function(x) {
                              ((x[2]-x[1])/15 + (x[3]-x[2])/30 + (x[4]-x[3])/70) / 3   # average
slope along x
}
null_slope <- apply(null, 1, slope);
a_slope <- apply(a, 1, slope);
b_slope <- apply(b, 1, slope);
ab_slope <- apply(ab, 1, slope);
print(c("median slope of null = ", median(null)));
print(c("median slope of a = ", median(a)));
print(c("median slope of b = ", median(b)));
print(c("median slope of ab = ", median(ab)));
print(wilcox.test(a_slope, b_slope));
print(wilcox.test(a_slope, ab_slope));
print(wilcox.test(b_slope, ab_slope));
# statistical interaction:
i5 <- null[,1] - a[,1] - b[,1] + ab[,1];
print(c("median interaction between a and b at t=5: ", median(i5)));
```

```
print(wilcox.test(i5));

i20 <- null[,2] - a[,2] - b[,2] + ab[,2];
print(c("median interaction between a and b at t=20: ", median(i20)));
print(wilcox.test(i20));

i50 <- null[,3] - a[,3] - b[,3] + ab[,3];
print(c("median interaction between a and b at t=50: ", median(i50)));
print(wilcox.test(i50));

i120 <- null[,4] - a[,4] - b[,4] + ab[,4];
print(c("median interaction between a and b at t=120: ", median(i120)));
print(wilcox.test(i120));
```
